# Hyperfibrinolysis During Caesarean Section and Vaginal Delivery: A Prospective Cross-Sectional Study in the Delivery Room

**DOI:** 10.3390/jcm15010027

**Published:** 2025-12-20

**Authors:** Philipp Zoidl, Gabriel Honnef, Michael Eichinger, Michael Eichlseder, Lioba Heuschneider, Sascha Hammer, Nikolaus Schreiber, Florian Prüller, Eva Christine Weiss, Bettina Amtmann, Helmar Bornemann-Cimenti

**Affiliations:** 1 Division of Anaesthesiology and Intensive Care Medicine 1, Department of Anaesthesiology and Intensive Care, Medical University of Graz, Auenbruggerplatz 5, 8036 Graz, Austria; 2 Division of Anaesthesiology and Intensive Care Medicine 2, Department of Anaesthesiology and Intensive Care, Medical University of Graz, Auenbruggerplatz 5, 8036 Graz, Austria; 3 Clinical Institute for Medical and Chemical Laboratory Diagnostics (KIMCL), University Hospital Graz, Auenbruggerplatz, 8036 Graz, Austria; 4 Department of Obstetrics and Gynecology, Medical University of Graz, Auenbruggerplatz 14, 8036 Graz, Austria

**Keywords:** hyperfibrinolysis, birth, Caesarean sectio, vaginal birth, coagulation disorder

## Abstract

**Introduction**: Postpartum hemorrhage remains a leading cause of maternal morbidity and mortality worldwide. While antifibrinolytic agents such as tranexamic acid are effective in treating established postpartum hemorrhage, the benefit of prophylactic tranexamic acid remains debated. The presence and frequency of early postpartum hyperfibrinolysis during routine childbirth have not been thoroughly investigated. **Material & Methods**: This prospective observational study was registered on ClinicalTrials.gov (NCT05975112) and conducted at the Medical University Hospital Graz between June 2023 and June 2024. Blood samples were collected from 413 women immediately after umbilical cord clamping; 379 were included in the analysis—291 undergoing Caesarean section and 88 vaginal delivery. Hyperfibrinolysis was assessed using thromboelastography and defined as an LY30 value > 8%. Additional coagulation parameters—including fibrinogen, D-dimer, activated partial thromboplastin time, and prothrombin time—were measured. Correlation analyses between viscoelastic and conventional parameters were performed using Pearson’s correlation coefficients. **Results**: No cases of clinically significant hyperfibrinolysis (LY30 > 8%) were observed. However, 15.5% of women showed elevated LY30 values (>0%). LY30 values were significantly higher in vaginal deliveries compared to Caesarean sections (*p* = 0.003). A moderate correlation between maximum amplitude (MA) and fibrinogen was observed (r = 0.52), strongest in vaginal deliveries (r = 0.65). Other correlations were weak or negligible. **Conclusions**: Clinically relevant hyperfibrinolysis was not observed immediately postpartum in women without hemorrhage. These findings are consistent with current guidelines recommending tranexamic acid for therapeutic rather than routine prophylactic use. Viscoelastic testing may be useful for rapid assessment in early-stage bleeding. Further research should explore fibrinolytic activity later in the postpartum period.

## 1. Introduction

Postpartum hemorrhage (PPH) remains a leading cause of maternal morbidity and mortality worldwide [1,2,3]. The World Health Organization (WHO) defines PPH as blood loss ≥ 500 mL/24 h after birth and a severe PPH as a blood loss of ≥1000 mL/24 h after birth [4]. 14 million women are affected every year, with approximately 70,000 maternal deaths worldwide per year. This represents 25% of maternal deaths during birth [4,5,6,7].

Despite efforts to improve prevention and treatment, an increasing incidence of PPH has been observed in recent years [4,7]. Reasons for this increasing incidence of PPH are higher rates in uterine atony, implantation disorders and rising rates of vaginal surgery as well as Caesarean sections with consecutive increased primary blood loss [8].

According to the current guidelines, the major intervention in the postpartum period is drug prophylaxis with uterotonics such as oxytocin (3–5 IU iv) or carbetocin (200 mcg iv) [9,10].

In addition to uterine atony, trauma, and placental abnormalities, disturbances of hemostasis—particularly hyperfibrinolysis—may contribute to the progression of PPH. Early identification of women at risk remains a challenge [1]. Tranexamic acid, an antifibrinolytic agent, has proven effective in reducing bleeding-related mortality in parturients with PPH. The WOMAN trial demonstrated that Tranexamic acid (TXA) reduces death due to bleeding in women with PPH, supporting its recommendation for PPH treatment [2].

Given the benefits of early TXA administration in treating PPH, research has focused on its prophylactic use during childbirth. Several studies have investigated the efficacy of prophylactic TXA in reducing blood loss during elective Caesarean sections [2,11]. While some trials demonstrate significantly reduced blood loss with prophylactic TXA in Caesarean sections [11], concerns remain regarding methodological limitations and potential thromboembolic events [12,13]. A large randomized controlled trial found that prophylactic TXA resulted in a significantly lower incidence of estimated blood loss greater than 1000 mL or red-cell transfusion in women undergoing Caesarean sections [13]. The TRAAP trial investigated TXA for preventing PPH following vaginal delivery but did not show a reduction in PPH [12].

Thromboelastography (TEG) is an important tool in the diagnosis and management of PPH. It offers a comprehensive assessment of hemostatic function by evaluating the viscoelastic properties of whole blood. As a point-of-care test, TEG provides a rapid, global view of coagulation, allowing for differentiation between various causes of bleeding, including factor deficiencies, hyperfibrinolysis, and medication-induced coagulopathies [14]. In the context of postpartum hemorrhage, where timely diagnosis and targeted intervention are vital, TEG can guide hemostatic resuscitation strategies and optimize transfusion algorithms [15,16,17]. Of particular interest is therefore the lysis after 30 min (LY30), which indicates the percentage of clot lysis (breakdown) 30 min after reaching the maximum clotting strength (MA). It reflects the degree of fibrinolysis occurring in the blood sample. LY30 values > 8% indicate hyperfibrinolysis [18].

However, it remains unclear how frequently hyperfibrinolysis occurs immediately after delivery. The present study addresses this question by quantifying the incidence of early postpartum hyperfibrinolysis and exploring the correlation between viscoelastic test results and conventional coagulation parameters.

Therefore, the primary aim of this study was to quantify the incidence of hyperfibrinolysis during vaginal delivery and Caesarean section immediately after cutting the umbilical cord. The secondary objective was to determine the correlations between the results of the TEG measurement and the measured quantitative coagulation parameters.

## 2. Methods

### 2.1. Study Design and Patient Population

This prospective observational study was conducted in the delivery room of the Department of Obstetrics and Gynecology of the Medical University Hospital Graz which the regional maximum care provider with over 3300 births per year. The Caesarean section rate in 2024 was 32.9%. Most of the women giving birth are of Caucasian ethnicity.

Women who delivered their child between 1 June 2023 and 28 June 2024 either vaginally or by Caesarean section were included in the study.

The study follows the STROBE guidelines for observational research [19].

### 2.2. Inclusion and Exclusion Criteria

Inclusion criteria:

All women aged 18 or older who planned to deliver their baby by Caesarean sectio or vaginal delivery at the delivery room at the Department of Obstetrics and Gynecology at the Medical University Hospital of Graz.

Exclusion criteria:

Exclusion criteria were emergency Caesarean sections and any known history of thrombocytopathy or coagulation disorders. In addition, women receiving medications known to affect platelet function or coagulation—such as acetylsalicylic acid (ASA), or antiplatelet agents including clopidogrel, prasugrel, or ticagrelor—were excluded. Patients who did not provide written informed consent were also not eligible for participation.

### 2.3. Measurements

Maternal blood was drawn immediately after the umbilical cord was cut and analyzed using thrombelastography (TEG 5000, Haemonetics Corporation, Boston, MA, USA). Blood samples were immediately transported to the laboratory, and analysis was initiated within 20 min of sampling in accordance with the manufacturer’s protocol.

In addition to viscoelastic testing, the following quantitative coagulation parameters were measured: fibrinogen concentration (reference range: 210–400 mg/dL), D-dimer levels (DD) (reference range: <500 ng/mL), activated partial thromboplastin time (APTT) (reference range: 26–36 s), and prothrombin time (PT) (reference range: 70–120% as well as Hb-Levels (12.0–15.3 g/dL).

In addition, clinical data and patient characteristics such as the mother’s age and mode of birth were recorded.

### 2.4. Sample Size Calculation

To the best of our knowledge, no study on the incidence of hyperfibrinolysis in women undergoing routine childbirth immediately after cord clamping has been conducted at the time of planning the study. Therefore, we choose a sample of convenience of minimum 300 subjects per group. We found this number to be reasonably included within half a year.

Due to limited laboratory resources, we planned the following procedure for this study: Blood sampling at the first three Caesarean sections per day and at the first three vaginal deliveries after the start of the day shift.

### 2.5. Statistical Analysis

Data was extracted from the laboratory database and merged into a database using Microsoft Excel (Microsoft Office 2016, Redmond, WA, USA). Demographic data were presented as median (25th to 75th percentile), or number (n) and percentages (%), as appropriate. Analyses were conducted using IBM SPSS statistics 27 (IBM, Redmond, WA, USA). The significance level for all analyses was 0.05.

The proportion of hyperfibrinolysis is reported using descriptive statistics. Differences between independent groups were analyzed using the non-parametric Mann–Whitney U test. Differences in patient characteristics were calculated using the independent samples *t*-test (age) and the Mann–Whitney U test, as appropriate. Linear associations between viscoelastic parameters and conventional coagulation markers were assessed using Pearson’s correlation coefficients (r) with corresponding two-sided *p*-values and 95% confidence intervals. Scatter plots were visually inspected to assess approximate linearity and to exclude major outliers before applying Pearson’s correlation. We acknowledge that this approach primarily captures linear relationships and may not fully reflect potential non-linear associations.

Analyses were performed both for the total cohort and stratified by predefined subgroups; cases with incomplete data sets were excluded.

### 2.6. Objectives

The primary objective of this study was to measure and quantify the incidence of hyperfibrinolysis in women undergoing both vaginal delivery and Caesarean section at the earliest possible time point, immediately after clamping the umbilical cord. Hyperfibrinolysis was defined as an LY30 value greater than 8%. To evaluate whether there was a statistically significant difference in the proportion of cases with elevated LY30 values (>0%) between delivery modes, a Pearson’s chi-squared test was performed.

The secondary aim of this study was to evaluate the linear relationship between selected continuous viscoelastic parameters and conventional coagulation values, namely the relationship between R-time and APTT and the relationship between MA and fibrinogen. Pearson’s correlation coefficients were calculated for the total cohort and for subgroups by mode of delivery.

## 3. Results

### 3.1. Characteristics of the Study Subjects

**Figure 1:**
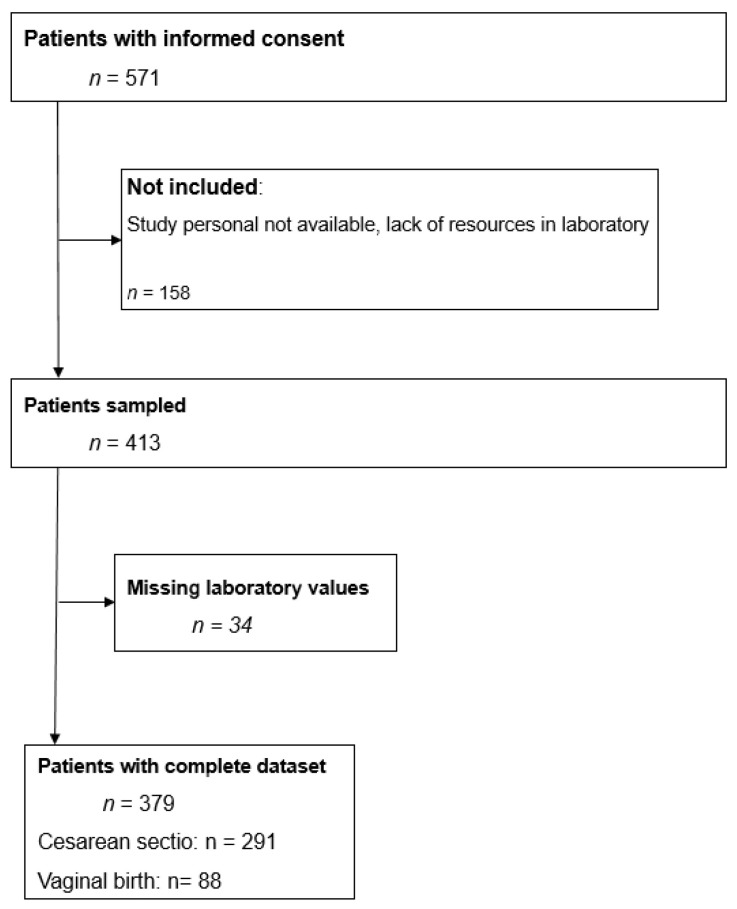
Study flow-chart.

571 parturients provided written informed consent, of whom 379 could be finally included (Figure 1). 291 gave birth by Caesarean section and 88 by vaginal delivery (Figure 1).

The median age of parturients was 32 years (IQR 29.2–35.8); 31 years (IQR 26.5–33.6) for vaginal deliveries and 32.6 years (IQR 29.7–36.4) for Caesarean sections.

### 3.2. Primary Outcome: Incidence of Hyperfibrinolysis

All variables met the assumptions of continuity and approximate interval scale. Scatter plots were visually inspected to assess linearity.

No cases of hyperfibrinolysis were observed. However, an elevated LY30 value (LY30 > 0) was observed in 51 out of 328 cases (15.5%).

Among women undergoing Caesarean section, 41 out of 291 cases (14.1%) showed LY30 > 0, compared to 10 out of 88 cases (11.4%) following vaginal delivery. The difference was not statistically significant (*p* = 0.56) (Table 1).

None of the parturients received TXA.

No cases of clinically significant hyperfibrinolysis (LY30 > 8%) were observed among the 379 parturients. Using the rule of three, the upper bound of the one-sided 95% confidence interval for a zero-event outcome in this sample is approximately 0.8%, indicating that clinically relevant systemic hyperfibrinolysis at this early postpartum time point is unlikely to occur at a prevalence above about 1%.

### 3.3. Secondary Outcomes: Correlation Analyses

R-time showed a weak positive correlation with APTT in the total cohort (r = 0.15, *p* > 0.05; 95% CI 0.04–0.25).

**Figure 2:**
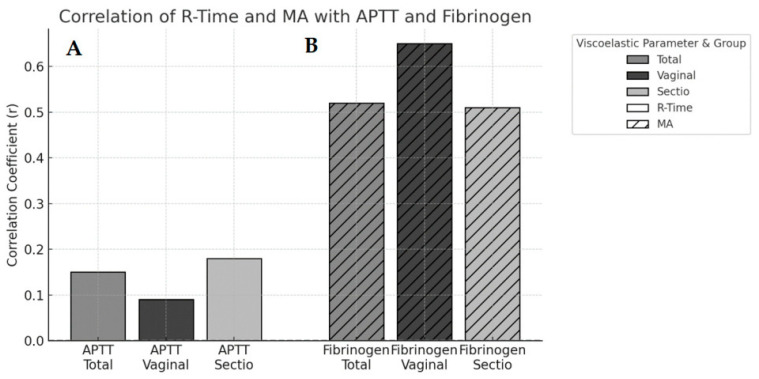
Pearson correlations between viscoelastic and conventional parameters. Panel A shows the correlation between R-time and activated partial thromboplastin time (aPTT), and Panel B shows the correlation between maximum amplitude (MA) and fibrinogen concentration.

Maximum amplitude (MA) demonstrated a moderate positive correlation with fibrinogen concentration in the total cohort (r = 0.52, *p* < 0.001; 95% CI 0.45–0.58). This association was strongest in the vaginal delivery subgroup (r = 0.65, *p* < 0.001; 95% CI 0.49–0.77) and remained moderate in women undergoing Caesarean section (r = 0.51, *p* < 0.001; 95% CI 0.43–0.58) (Table 2, Figure 2).

## 4. Discussion

In this prospective observational single-center study including 379 parturients giving routine birth via either Caesarean section or vaginal birth, no case of systemic hyperfibrinolysis was observed.

To our knowledge, there are no studies in which TEG has been investigated in parturients who did not have PPH. In a study from 2020, no hyperfibrinolysis was found in 118 TEGs performed for manifest PPH [20]. In another study from 2018, hyperfibrinolysis was found in 35 out of 167 women giving birth with PPH, accounting for 23% [21].

From a statistical perspective, the absence of hyperfibrinolytic events in 379 women does not imply that the true risk is zero, but the rule of three indicates that clinically relevant systemic hyperfibrinolysis is unlikely to occur with a prevalence greater than approximately 0.8% at this earliest postpartum time point. This supports the conclusion that systemic hyperfibrinolysis is rare immediately after delivery in women without hemorrhage, while not excluding rare events below this threshold.

Although no cases of clinically significant hyperfibrinolysis were observed, 15.5% of women showed LY30 values above 0%. These mildly elevated LY30 values, which remained well below the conventional hyperfibrinolysis threshold of 8%, may reflect early physiological fibrinolytic rebalancing rather than pathological systemic fibrinolysis. During pregnancy, fibrinolytic activity is suppressed, whereas postpartum a gradual restoration of fibrinolytic capacity occurs as part of the return towards the non-pregnant haemostatic state. This process is likely to be driven by local fibrin degradation at the uteroplacental site and may initially manifest as low-grade or localized fibrinolytic activation without systemic hyperfibrinolysis or clinical bleeding. Our findings are therefore consistent with the concept of a controlled, physiological increase in fibrinolytic potential after delivery, as described in previous studies of postpartum haemostatic changes [22,23,24].

While physiological activation of fibrinolysis is a normal local process during and after childbirth potentially due to tissue damage, the absence of systemic hyperfibrinolysis in this cohort of women suggests that the fibrinolytic process remains localized to the uterus in the absence of a major bleeding event [22,23]. Other studies have indicated that elevated fibrinolytic potential can be a risk factor for PPH, but it does not inevitably lead to clinical PPH [1]. Identification of patients at risk for PPH before birth is difficult and only possible with a profound risk factor such as a placenta accreta situation [3].

During pregnancy, the hemostatic system undergoes significant changes, including a decrease in physiological anticoagulants and impaired fibrinolytic activity. This creates a tendency toward coagulation, which is balanced by an increase in fibrinolysis postpartum. However, in some cases, this balance can be disrupted, leading to either excessive coagulation or hyperfibrinolysis [24].

Thromboelastography, particularly the TEG 5000 system, has known limitations in the detection of early or low-grade fibrinolytic activation. Compared with ROTEM, which offers fibrinolysis-specific channels such as APTEM or FIBTEM to unmask fibrinolytic activity, TEG relies primarily on clot lysis parameters derived from the native assay. As a result, subtle or early fibrinolytic changes—especially when localized to the uteroplacental compartment—may not be detected. This limitation is particularly relevant in obstetrics, where fibrinolysis may initially be compartmental rather than systemic. Nevertheless, LY30 remains the most commonly used and clinically validated fibrinolysis parameter in TEG-based algorithms, with established thresholds for clinically relevant hyperfibrinolysis. An LY30 value > 8% has been consistently associated with severe systemic fibrinolysis and adverse bleeding outcomes. Given the exploratory nature of this study and the absence of postpartum hemorrhage in our cohort, LY30 was considered appropriate to detect clinically meaningful systemic hyperfibrinolysis rather than subclinical or localized fibrinolytic activity [18]. Therefore, while our findings do not exclude early or localized fibrinolytic activation undetectable by TEG, they suggest that clinically significant systemic hyperfibrinolysis is unlikely to occur immediately after delivery in women without bleeding. Future studies combining serial sampling with more fibrinolysis-sensitive viscoelastic assays may further clarify postpartum fibrinolytic dynamics.

Further research is needed to refine diagnostic criteria for hyperfibrinolysis and to optimize the use of viscoelastic testing in the peripartum setting and determination of the optimal use of viscoelastic tests like TEG to assess peripartal coagulopathy seems to be necessary. Monitoring changes in the coagulation mechanism during the postpartum period is fundamental, and clinicians should remain vigilant regarding the possibility of fibrinolytic hyperactivity. Some studies have shown a correlation between altered values in the viscoelastic test procedures and quantitative coagulation values. For example, Huissoud C et al. showed that maximum clot formation (MCF) correlates significantly with fibrinogen levels (r = 0.82, *p* < 0.001), a finding we were also able to confirm, showing a correlation coefficient of r = 0.52 for MA in TEG [25,26].

While comparisons between Caesarean section and vaginal delivery were included for exploratory purposes, we acknowledge that fibrinolytic differences within vaginal deliveries—particularly in the presence of bleeding, tissue trauma, or prolonged labor—may be more clinically informative. Future studies should specifically address within-group variability among vaginal deliveries in larger cohorts and in the context of postpartum hemorrhage.

Identification of coagulopathy by viscoelastic point-of-care testing or conventional laboratory assays can be helpful in guiding management of PPH and preventing deleterious maternal outcomes [27].

Further research on the rate and role of hyperfibrinolysis occurring later in the postpartum period seems necessary as well as the extent of local hyperfibrinolysis. These studies should carefully consider patient selection and the mode of delivery. A comparison of fibrinolytic parameters between women with and without a significant postpartum hemoglobin decline could provide additional clinical insight, but the low number of such cases in our cohort precluded meaningful analysis. This question should be addressed in studies specifically designed to include bleeding outcomes.

## 5. Limitations

A key limitation of this study is the very early timing of blood sampling, which was performed immediately after umbilical cord clamping. While this approach allowed standardized and uniform data collection at the earliest possible postpartum time point, it may underestimate fibrinolytic activity that develops later in the postpartum period. Consequently, our findings cannot exclude delayed or evolving systemic hyperfibrinolysis occurring minutes to hours after delivery, particularly in the context of ongoing bleeding or secondary uterine atony.

Another important limitation is the marked imbalance in group sizes, with substantially fewer women giving birth vaginally (n = 88) compared with those undergoing Caesarean section (n = 291). This reflects the practical difficulty of prospectively recruiting and obtaining informed consent in unplanned vaginal deliveries. As a consequence, the statistical power to detect differences in LY30 and to estimate correlations in the vaginal delivery subgroup is limited, and subgroup results should therefore be interpreted with caution. Our main conclusions are primarily based on the total cohort, in which the precision of estimates is higher.

Finally, our correlation analysis was based on Pearson’s correlation coefficients, which quantify linear associations. Although visual inspection of scatter plots did not suggest marked non-linearity, more complex or subtle non-linear relationships between viscoelastic parameters and coagulation markers may not be fully captured by this approach.

## 6. Conclusions

In this prospective observational laboratory study conducted in the delivery room, no cases of hyperfibrinolysis were observed in 413 births immediately after delivery. A rate of LY30 values above 0 of 11% (vaginal delivery) and 14% (Caesarean section) was found. This study suggests that clinically relevant systemic hyperfibrinolysis is rare in the immediate postpartum period and that viscoelastic parameters, particularly MA, correlate meaningfully with conventional markers such as fibrinogen.

## Figures and Tables

**Table 1:** Demographic data and baseline characteristics.

	Total (n = 379)	Vaginal Delivery (n = 88)	Caesarean Section (n = 291)	*p*-Value
Age (year)	32.2 (IQR 29.2–35.8)	31.0 (IQR 26.5–33.7)	32.6 (IQR 29.7–36.4)	0.0001
Gestation age (week + day)	38 + 6 (IQR 37 + 6–39 + 5)	40 + 1 (IQR 39 + 4–40 + 4)	38 + 4 (IQR 37 + 4–39 + 0)	<0.0001
BMI before pregnancy	28.2 (IQR 24.9–32.0)	27.1 (IQR 24.5–29.8)	28.7 (IQR 25.0–32.9)	0.023
Gemini (n=)	3	0	3	
Preterm birth (n=)	20	0	20	
Red blood cells				
Hb (g/dL) immediately postpartum	11.3 (IQR 10.6–12.2)	12.2 (IQR 10.3–12.9)	11.3 (IQR 10.6–12.0)	
Hb-drop > 2 (n=) at delivery room discharge	11	4	7	
TEG				
R-Time (s)	230.00 (IQR 200.00–275.00)	245.00 (IQR 211.25–283.75)	225.00 (IQR 195.00–270.00)	0.068
MA (mm)	72.3 (IQR 70.1–74.2)	71.8 (IQR 69.95–73.95)	72.3 (IQR 70.10–74.20)	0.467
LY30 (%)	0	0	0	0.489
Quantitative coagulation values				
APTT (s)	25.9 (IQR 24.1–28.0)	25.35 (IQR 23.77–27.42)	26.00 (IQR 24.40–28.10)	0.029
PT	107.0 (IQR 99.0–114.0)	109.0 (IQR 100.0–115.0)	106.0 (IQR 98.0–112.0)	0.07
DD	2.47 (IQR 1.70–3.84)	2.80 (IQR 1.94–4.44)	2.36 (IQR 1.58–3.32)	0.002
Fibrinogen	458.00 (IQR 402.50–516.00)	490.50 (IQR 436.75–566.75)	449.00 (IQR 98.00–112.00)	<0.0001

**Table 2:** Pearson Correlation Summary Table.

Viscoelastic Parameter	Coagulation Marker	Cohort	r	95% CI	*p*-Value
R-time	aPTT	Total	0.15	0.04–0.25	>0.05
R-time	aPTT	Caesarean section	0.19	0.07–0.31	<0.01
R-time	aPTT	Vaginal delivery	0.04	−0.17–0.25	>0.05
MA	Fibrinogen	Total	0.52	0.45–0.58	<0.001
MA	Fibrinogen	Caesarean section	0.51	0.49–0.77	<0.001
MA	Fibrinogen	Vaginal delivery	0.65	0.43–0.58	<0.001

## Data Availability

Available upon reasonable request.

## References

[1] Gruneberg D., Braun P., Schöchl H., Nachtigall-Schmitt T., von der Forst M., Tourelle K., Dietrich M., Wallwiener M., Wallwiener S., Weigand M.A. (2023). Fibrinolytic potential as a risk factor for postpartum hemorrhage. Front. Med..

[2] Collaborators W.T. (2017). Effect of early tranexamic acid administration on mortality, hysterectomy, and other morbidities in women with post-partum haemorrhage (WOMAN): An international, randomised, double-blind, placebo-controlled trial. Lancet.

[3] Bienstock J.L., Eke A.C., Hueppchen N.A. (2021). Postpartum Hemorrhage. N. Engl. J. Med..

[4] World Health Organization (2023). A Roadmap to Combat Postpartum Haemorrhage Between 2023 and 2030.

[5] Sheldon W., Blum J., Vogel J., Souza J., Gülmezoglu A., Winikoff B. (2014). on behalf of the WHO Multicountry Survey on Maternal and Newborn Health Research Network. Postpartum haemorrhage management, risks, and maternal outcomes: Findings from the World Health Organization Multicountry Survey on Maternal and Newborn Health. BJOG Int. J. Obstet. Gynaecol..

[6] Li S., Gao J., Liu J., Hu J., Chen X., He J., Tang Y., Liu X., Cao Y., Liu X. (2021). Incidence and Risk Factors of Postpartum Hemorrhage in China: A Multicenter Retrospective Study. Front. Med..

[7] Corbetta-Rastelli C.M., Friedman A.M., Sobhani N.C., Arditi B., Goffman D., Wen T. (2023). Postpartum Hemorrhage Trends and Outcomes in the United States, 2000–2019. Obstet. Gynecol..

[8] Kramer M., Dahhou M., Vallerand Dm Liston R., Joseph K.S. (2011). Risk Factors for Postpartum Hemorrhage: Can We Explain the Recent Temporal Increase?. J. Obstet. Gynaecol. Can..

[9] DGGG (2022). S2k-Leitlinie Peripartale Blutungen, Diagnostik und Therapie. https://register.awmf.org/de/leitlinien/detail/015-063.

[10] Gallos I.D., Papadopoulou A., Man R., Athanasopoulos N., Tobias A., Price M.J., Williams M.J., Diaz V., Pasquale J., Chamillard M. (2018). Uterotonic agents for preventing postpartum haemorrhage: A network meta-analysis. Cochrane Database Syst. Rev..

[11] Lakshmi S.D., Abraham R. (2016). Role of Prophylactic Tranexamic Acid in Reducing Blood Loss during Elective Caesarean Section: A Randomized Controlled Study. J. Clin. Diagn. Res..

[12] Sentilhes L., Winer N., Azria E., Sénat M.V., Le Ray C., Vardon D., Perrotin F., Desbrière R., Fuchs F., Kayem G. (2018). Tranexamic Acid for the Prevention of Blood Loss after Vaginal Delivery. N. Engl. J. Med..

[13] Sentilhes L., Sénat M.V., Le Lous M., Winer N., Rozenberg P., Kayem G., Verspyck E., Fuchs F., Azria E., Gallot D. (2021). Tranexamic Acid for the Prevention of Blood Loss after Cesarean Delivery. N. Engl. J. Med..

[14] Herkommer M., Geisler T. (2010). Verfahren zur Messung der Thrombozytenfunktion: Pro und kontra. Kardiol. Up2date.

[15] Karlsson O., Jeppsson A., Hellgren M. (2014). Major obstetric haemorrhage: Monitoring with thromboelastography, laboratory analyses or both?. Int. J. Obstet. Anesth..

[16] Ekelund K., Hanke G., Stensballe J., Wikkelsøe A., Albrechtsen C.K., Afshari A. (2015). Hemostatic resuscitation in postpartum hemorrhage—A supplement to surgery. Acta Obstet. Et Gynecol. Scand..

[17] Hill J.S., Devenie G., Powell M. (2012). Point-of-Care Testing of Coagulation and Fibrinolytic Status during Postpartum Haemorrhage: Developing a Thrombelastography®-Guided Transfusion Algorithm. Anaesth. Intensive Care.

[18] Whiting D., DiNardo J.A. (2014). TEG and ROTEM: Technology and clinical applications. Am. J. Hematol..

[19] von Elm E., Altman D.G., Egger M., Pocock S.J., Gøtzsche P.C., Vandenbroucke J.P. (2007). The Strengthening the Reporting of Observational Studies in Epidemiology (STROBE) statement: Guidelines for reporting observational studies. Lancet.

[20] Arnolds D.E., Scavone B.M. (2020). Thromboelastographic Assessment of Fibrinolytic Activity in Postpartum Hemorrhage: A Retrospective Single-Center Observational Study. Anesth. Analg..

[21] Roberts I., Shakur H., Fawole B., Kuti M., Olayemi O., Bello A., Ogunbode O., Kotila T., Aimakhu C.O., Olutogun T. (2018). Haematological and fibrinolytic status of Nigerian women with post-partum haemorrhage. BMC Pregnancy Childbirth.

[22] Suarez C.R., Walenga J., Mangogna L.C., Fareed J. (1985). Neonatal and maternal fibrinolysis: Activation at time of birth. Am. J. Hematol..

[23] Takeda S., Takeda J., Makino S., Schmolzer G. (2019). Hemostasis for Massive Hemorrhage during Cesarean Section. Recent Advances in Cesarean Delivery.

[24] Bremme K.A. (2003). Haemostatic changes in pregnancy. Best Pract. Res. Clin. Haematol..

[25] Amgalan A., Allen T., Othman M., Ahmadzia H.K. (2020). Systematic review of viscoelastic testing (TEG/ROTEM) in obstetrics and recommendations from the women’s SSC of the ISTH. J. Thromb. Haemost..

[26] Huissoud C., Carrabin N., Benchaib M., Fontaine O., Levrat A., Massignon D., Touzet S., Rudigoz R.C., Berland M. (2009). Coagulation assessment by rotation thrombelastometry in normal pregnancy. Thromb. Haemost..

[27] McLintock C. (2020). Prevention and treatment of postpartum hemorrhage: Focus on hematological aspects of management. Hematology.

